# Lnc-PTCHD4-AS inhibits gastric cancer through MSH2-MSH6 dimerization and ATM-p53-p21 activation

**DOI:** 10.18632/aging.205329

**Published:** 2023-11-27

**Authors:** Jingyun Wang, Yang Mi, Xiangdong Sun, Xia Xue, Huanjie Zhao, Mengfei Zhang, Baitong Hu, Ihtisham Bukhari, Pengyuan Zheng

**Affiliations:** 1 Henan Key Laboratory for Helicobacter pylori and Microbiota and GI Cancer, Marshall Medical Research Center, Fifth Affiliated Hospital of Zhengzhou University, Zhengzhou 450000, China; 2 Academy of Medical Science, Zhengzhou University, Zhengzhou 450000, China; 3 Department of Gastroenterology, Fifth Affiliated Hospital of Zhengzhou University, Zhengzhou 450000, China

**Keywords:** gastric cancer, long non-coding RNA PTCHD4-AS, DNA mismatch repair proteins, MSH2-MSH6, cisplatin

## Abstract

Conserved long non-coding RNAs (lncRNAs) have not thoroughly been studied in many cancers, including gastric cancer (GC). We have identified a novel lncRNA PTCHD4-AS which was highly conserved between humans and mice and naturally downregulated in GC cell lines and tissues. Notably, PTCHD4-AS was found to be transcriptionally induced by DNA damage agents and its upregulation led to cell cycle arrest at the G2/M phase, in parallel, it facilitated the cell apoptosis induced by cisplatin (CDDP) in GC. Mechanistically, PTCHD4-AS directly bound to the DNA mismatch repair protein MSH2-MSH6 dimer, and facilitated the binding of dimer to ATM, thereby promoting the expression of phosphorylated ATM, p53 and p21. Here we conclude that the upregulation of PTCHD4-AS inhibits proliferation and increases CDDP sensitivity of GC cells via binding with MSH2-MSH6 dimer, activating the ATM-p53-p21 pathway.

## INTRODUCTION

Long noncoding RNAs (lncRNAs) are a type of transcripts longer than 200 nucleotides without encoding any functional protein [1, 2]. LncRNAs widely regulate various physiological and pathological processes, such as embryonic development, cell differentiation, cell proliferation, apoptosis, tumorigenesis and drug resistance [3–5]. During the evolutionary process from lower to higher organisms, only a tiny fraction of lncRNA showed evolutionary conservation [6–8], and some of the conserved lncRNAs are known to play an essential role in tumor development, progression, and treatment [9–12]. For instance, lncRNA MALAT1 is conserved between humans and mice, and it is aberrantly expressed in a variety of tumors [13], such as lung cancer [14], breast cancer [15], glioma [16] and colon cancer [17]. It plays an oncogenic role [18], serves as a target for chemosensitization and radiosensitization of cancer cells [13]. Similarly, lncRNA ARA with a conserved sequence in primates [19] is known to significantly increase the sensitivity of cancer cells to drugs and inhibit the development of breast and hepatocellular carcinoma by activating MAPK signaling and metabolic pathways [20]. Similarly, lncRNA HOTAIR conserved between humans and mice can inhibit DNA damage repair and increases apoptosis through p53, thus suppressing tumor cell growth [21, 22].

A large number of abnormally expressed lncRNAs have been found in gastric cancer (GC), and some may act as oncogenes or tumor suppressor [23–25]. However, there are few studies on conserved non-coding RNAs in GC. The interspecies conserved lncRNA H19 promotes GC proliferation by inhibiting p53 [26]. lncRNA HOTAIR also acts as a competitive endogenous RNA to promote proliferation and metastasis in gastric cancer [27, 28].

In this study, we obtained conserved lncRNA information between humans and mice by homologous sequence alignment. We selected a lncRNA encoded by the antisense strand of the PTCHD4 genes; thus, we named it PTCHD4-AS. This lncRNA was naturally downregulated in the gastric cancer tissues and several GC cell lines. We demonstrated through *in vitro* and *in vivo* experiments that up-regulation of PTCHD4-AS inhibited the proliferation of gastric cancer cells and increased CDDP-induced apoptosis via interacting with DNA mismatch repair proteins MSH2-MSH6 dimer and activating the ATM-p53-p21 pathway.

## RESULTS

### PTCHD4-AS was downregulated and transcriptionally induced by DNA damage in GC

**Figure 1:**
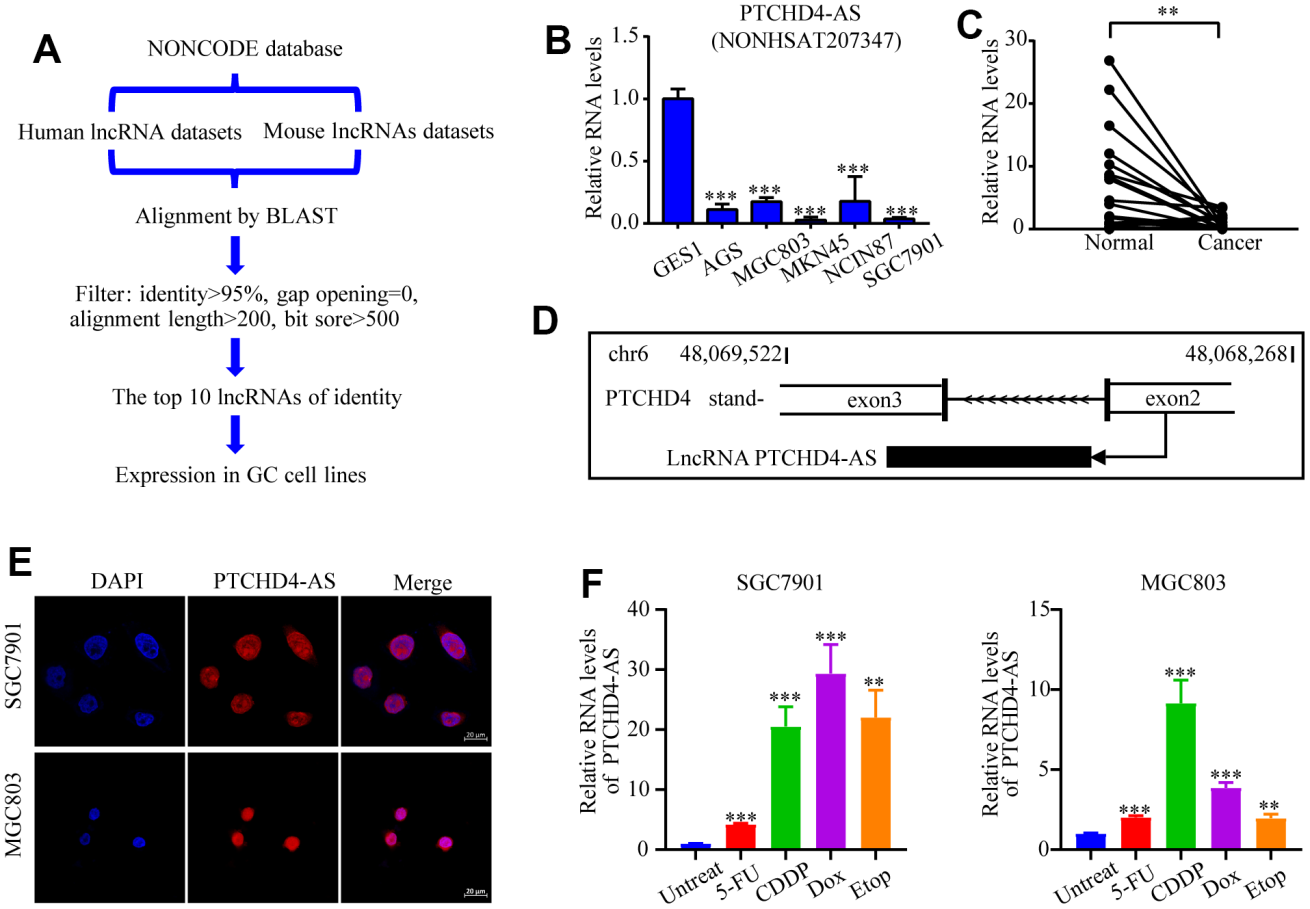
**Identification and characterization of PTCHD4-AS.** (**A**) Diagram of the screening strategy for conserved lncRNAs. (**B**) Relative expression levels of PTCHD4-AS in normal gastric epithelial cell line (GES-1) and GC cell lines (AGS, SGC7901, MGC803, MKN45 and NCI-N87). (**C**) Relative expression levels of PTCHD4-AS in paired GC tissues and adjacent normal gastric tissues. n=23. (**D**) Schematic diagram of the transcription of PTCHD4-AS. (**E**) Subcellular localization of PTCHD4-AS in SGC7901 and MGC803. DAPI labeled nuclei(blue); FISH probe labeled PTCHD4-AS (red), scale bar: 20 μm. (**F**) Relative expression of PTCHD4-AS after treatment of SGC7901 and MGC803 with the indicated concentrations of drugs for 48 h. Etop :20 μM, 5-FU :20 μM, CDDP: 10 μM, Dox:4 μM. All data are presented as mean ± SD, * *P* < 0.05, ** *P* < 0.01, *** *P* < 0.001.

To explore the role of highly conserved lncRNAs in GC, we obtained human and mouse non-coding RNA sequences from the NONCODE (V6) database [29], which contained 173,112 and 131,974 sequences, respectively. Homologous sequence alignment was conducted using the Basic Local Alignment Search Tool (BLAST) version 2.11.0 or higher [30] and selected the top 10 highly conserved lncRNAs for further analyses (Figure 1A and Supplementary Table 2).

Using RT-qPCR, we analyzed the levels of expression of these non-coding RNA in GC cell lines (Supplementary Figure 1A). We found that lncRNA NONHSAT207347, exhibited a notably low expression in many GC cell lines (AGS, SGC7901, MGC803, MKN45, and NCI-N87) as compared to the normal gastric epithelial cell line GES-1 (Figure 1B). Moreover, its expression was also reduced in GC tissues compared to paired normal tissues (Figure 1C). This lncRNA (NONHSAT207347) is a transcript located on chromosome 6 (48,068,268-48069522, GRCh38/hg38) and transcribed from the antisense strand of the protein-coding gene PTCHD4. Therefore, it was named PTCHD4-AS (Figure 1D). RACE assay confirmed its length is about 500 nucleotides (Supplementary Figure 1B), and CPAT [31] predicted that PTCHD4-AS does not encode a protein (Supplementary Figure 1C). Using FISH analysis and cytoplasmic and nuclear RNA fractionation assay, we observed that PTCHD4-AS is mainly localized in the nucleus (Figure 1E and Supplementary Figure 1D). Interestingly, we observed that the expression of PTCHD4-AS was significantly induced by various DNA-damaging agents (5-fluorouracil, cisplatin, doxorubicin, and etoposide) in GC cells (Figure 1F).

### PTCHD4-AS as a tumor suppressor in GC *in vitro*

**Figure 2:**
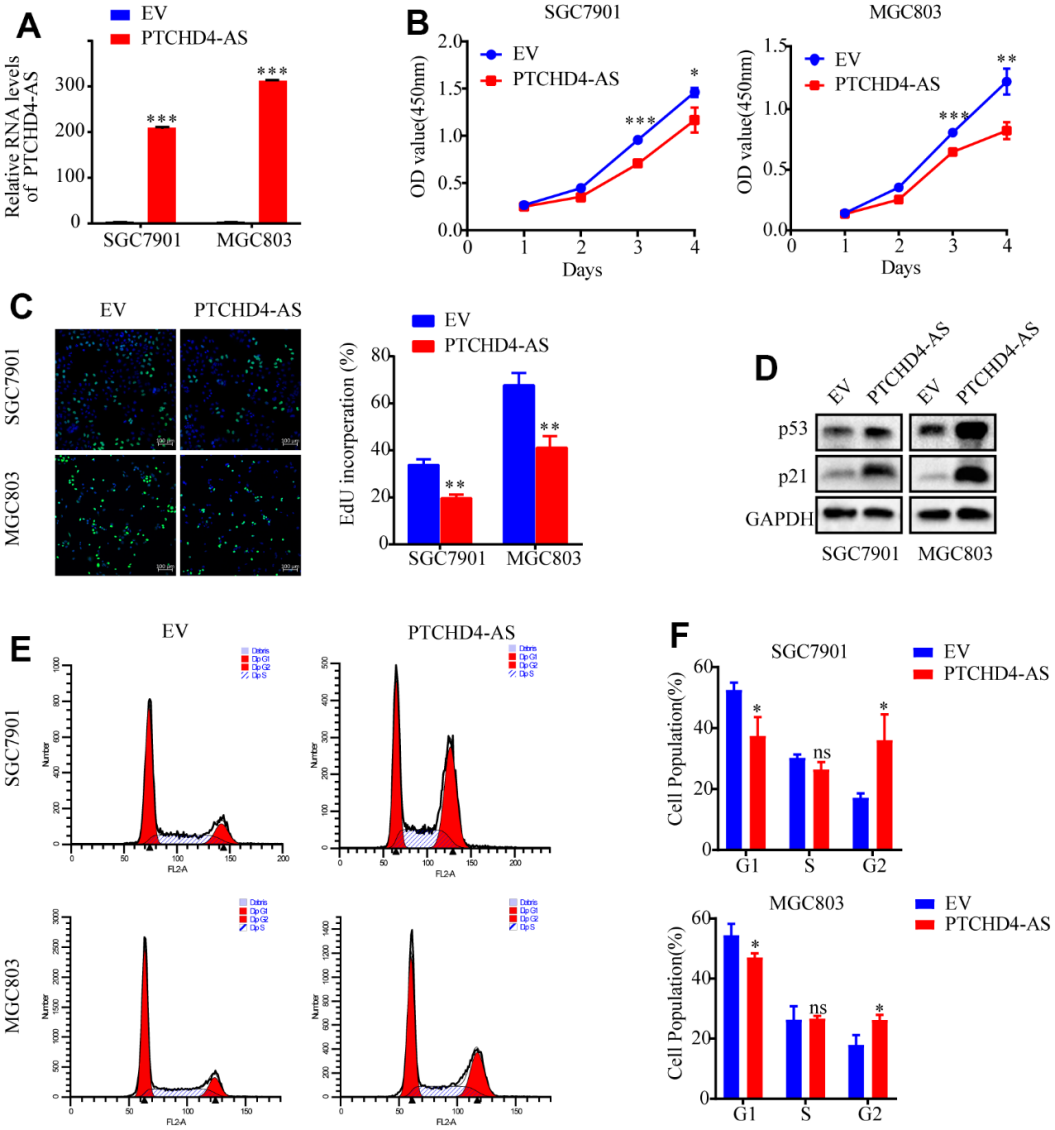
**Upregulation of PTCHD4-AS suppressed GC cell proliferation *in vitro*.** (**A**) Overexpression efficiencies of PTCHD4-AS was analyzed by qPCR in SGC7901 and MGC803. (**B**) Viability of SGC7901 and MGC803 cells stably overexpressing EV or PTCHD4-AS. (**C**) Representative images of EdU in SGC7901 and MGC803 cells stably overexpressing EV or PTCHD4-AS and statistical analysis of Edu-positive cells. (**D**) Expression of p53 and p21, key proteins of the cell cycle pathway, was detected in the indicated cells by western blotting. (**E**, **F**) Cell cycle analysis by FACS in SGC7901 and MGC803 cells stably expressing EV or PTCHD4-AS. (**A**–**F**) EV indicates cells transfected with blank vector and PTCHD4-AS indicates cells transfected with lncRNA PTCHD4-AS. Data are presented as mean ± SD, * *P* < 0.05, ** *P* < 0.01, *** *P* < 0.001.

In order to examine the impact of PTCHD4-AS on GC cell proliferation, we employed lentiviral transduction to overexpress PTCHD4-AS in SGC7901 and MGC803 cells (Figure 2A), showing that the overexpression of PTCHD4-AS significantly proliferation (Figure 2B, 2C) and colony formation capabilities of SGC7901 and MGC803 cells (Supplementary Figure 2A). The results of the Western blot analysis demonstrated that the overexpression of PTCHD4-AS led to an elevation in the protein levels of p53 and p21, which are recognized as crucial regulators of cell cycle arrest (Figure 2D). The flow cytometry confirmed that the overexpression of PTCHD4-AS resulted in cell cycle arrest, specifically at the G2 phase (Figure 2E, 2F). These findings indicate that PTCHD4-AS exhibit tumor suppressive properties in GC.

### PTCHD4-AS interaction with MSH2-MSH6 dimer in GC

**Figure 3:**
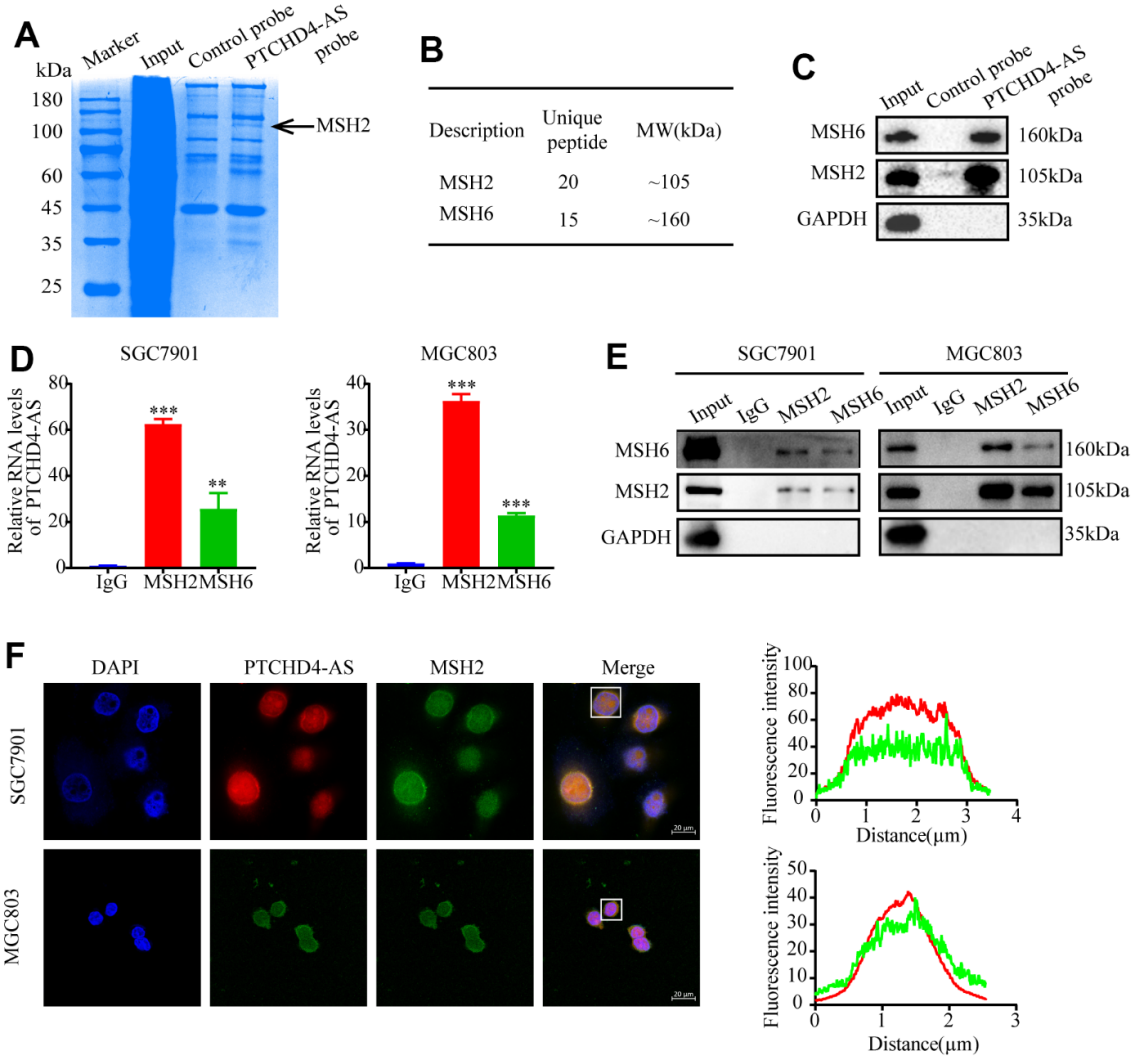
**PTCHD4-AS interacted with MSH2-MSH6 dimer in GC.** (**A**) SDS-PAGE of pull-down assay of whole cell lysates from SGC7901 cells using a biotin-labeled control/PTCHD4-AS probe showing PTCHD4-AS binding proteins. (**B**) Protein characterization of highly unique peptides by mass spectrometry. (**C**) Western blot analysis of the biotin-labeled probe pull-down eluate in (**A**). (**D**) RIP assay of anti-MSH2 or anti-MSH6 antibodies in SGC7901 and MGC803 cells confirmed that PTCHD4-AS interacted with the MSH2-MSH6 dimer. The levels of PTCHD4-AS in the precipitates were detected by RT-qPCR, and IgG was used as a negative control. (**E**) MSH2 and MSH6 protein levels in the precipitates were detected by Western blotting, and GAPDH was used as a loading control. (**F**) Representative confocal images and co-localization analysis of PTCHD4-AS (red) and MSH2 (green) in SGC7901 and MGC7901 cells. Data are presented as mean ± SD, * *P* < 0.05, ** *P* < 0.01, *** *P* < 0.001.

To identify the proteins that interact with PTCHD4-AS, we performed RNA pull-down assay using biotin-labeled PTCHD4-AS probes or control probes in GC cells (Figure 3A). Mass spectrometry analysis revealed that MSH2 and MSH6, two components of the DNA mismatch repair (MMR) pathway [32, 33], were potentially associated with PTCHD4-AS (Figure 3B and Supplementary Figure 2B). Western blot analysis confirmed that MSH2 and MSH6 were enriched in the PTCHD4-AS pull-down lysate (Figure 3C).

In order to verify the interaction of PTCHD4-AS and MSH2-MSH6 dimer, we performed the coIP assay using anti-MSH2 and anti-MSH6 antibodies in GC cells. We found that PTCHD4-AS was co-precipitated with both MSH2 and MSH6, indicating that it binds to the MSH2-MSH6 dimer (Figure 3D, 3E). Since PTCHD4-AS was mainly localized in the nucleus, as shown in Figure 1E and Supplementary Figure 1D, suggesting possible interaction with nuclear proteins such as MSH2 and MSH6. The main localization of MSH2 and MSH6 was detected within the nucleus of GC cells [34], suggesting a possible co-localization with PTCHD4-AS. Indeed, FISH and immunofluorescence co-staining showed that PTCHD4-AS and MSH2 were co-localized in the nucleus of GC cells (Figure 3F). These findings demonstrate that PTCHD4-AS directly interacts with MSH2-MSH6 dimer in GC.

### PTCHD4-AS suppressed GC proliferation via MSH2-MSH6 dimer

**Figure 4:**
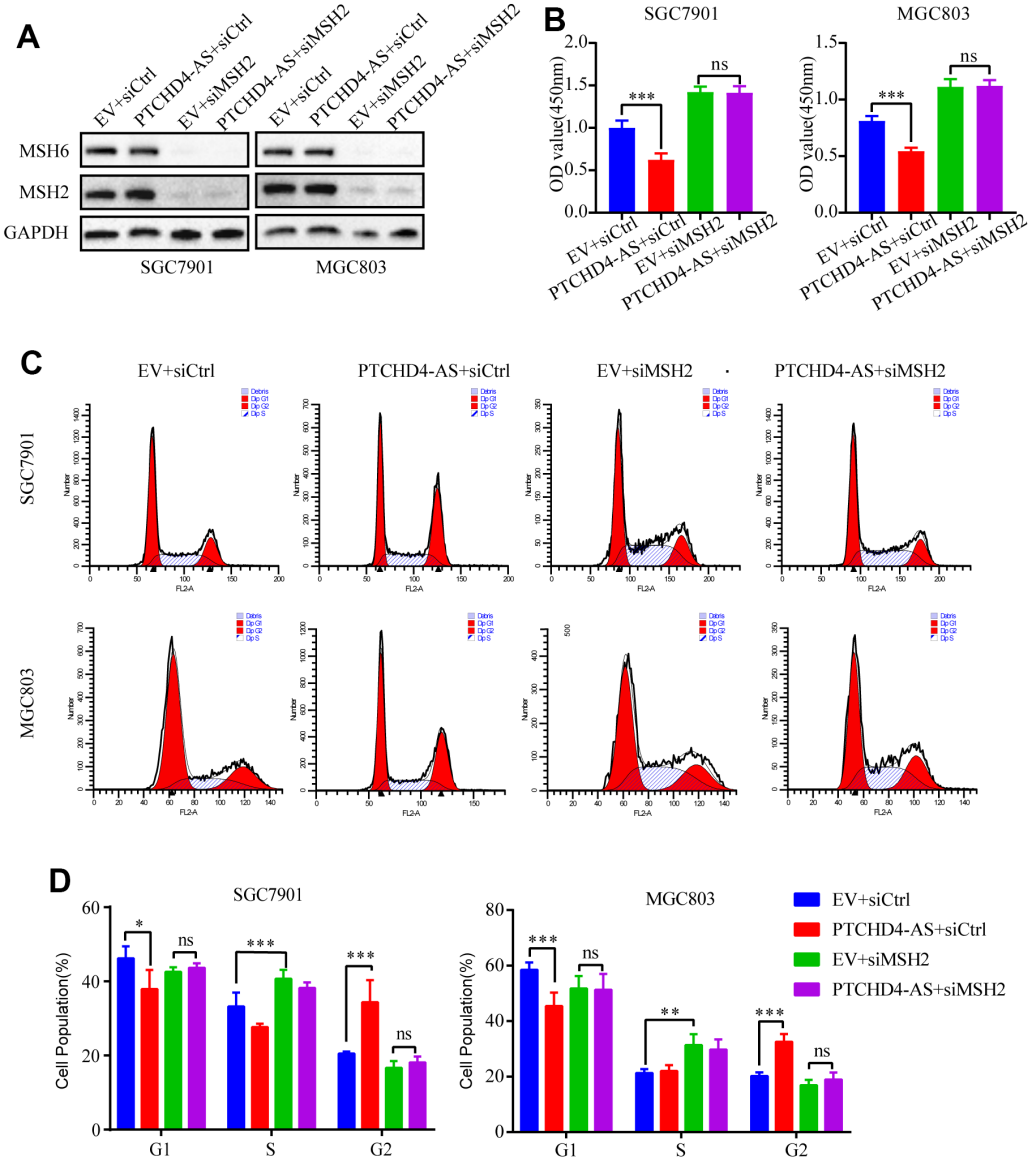
**PTCHD4-AS suppressed GC proliferation via MSH2-MSH6 dimer.** (**A**) Western blotting analysis of MSH2 and MSH6 protein levels in SGC7901 and MGC803 cells stably overexpressing EV or PTCHD4-AS after transduction with siCtrl or siMSH2 for 48 h. (**B**) Cell proliferation assays after transduction with siCtrl or siMSH2 for 72 h. (**C**, **D**) Cell cycle analysis after transduction with siCtrl or siMSH2 for 48 h. Data are presented as mean ± SD, * *P* < 0.05, ** *P* < 0.01, *** *P* < 0.001.

Previous studies observed that overexpression of MSH2 or MSH6 in cancer cells led to MMR-dependent activation of DNA damage signaling and cell cycle G2/M arrest [35–37]. Moreover, it has been reported that the stability of MSH6 protein depends on its interaction with MSH2 [34]. Consequently, we postulated that the PTCHD4-AS influence the activity of the MSH2-MSH6 dimer in GC cells. Therefore, we employed small interfering RNAs (siRNAs) to suppress the production of MSH2 in GC cells. It was observed that the downregulation of MSH2 led to a considerable decline in the protein expression of MSH6 (Figure 4A). Furthermore, this downregulation of MSH2 counteracted the inhibitory impact of PTCHD4-AS on the proliferation of GC cells (Figure 4B). Additionally, it was observed that the downregulation of MSH2 resulted in the reversal of the G2/M phase arrest caused by the overexpression of PTCHD4-AS (Figure 4C, 4D), suggesting that the tumor suppressive impact of PTCHD4-AS is facilitated by its interaction with the MSH2-MSH6 dimer.

### PTCHD4-AS activates the ATM-p53-p21 pathway by promoting MSH2-MSH6 dimerization

**Figure 5:**
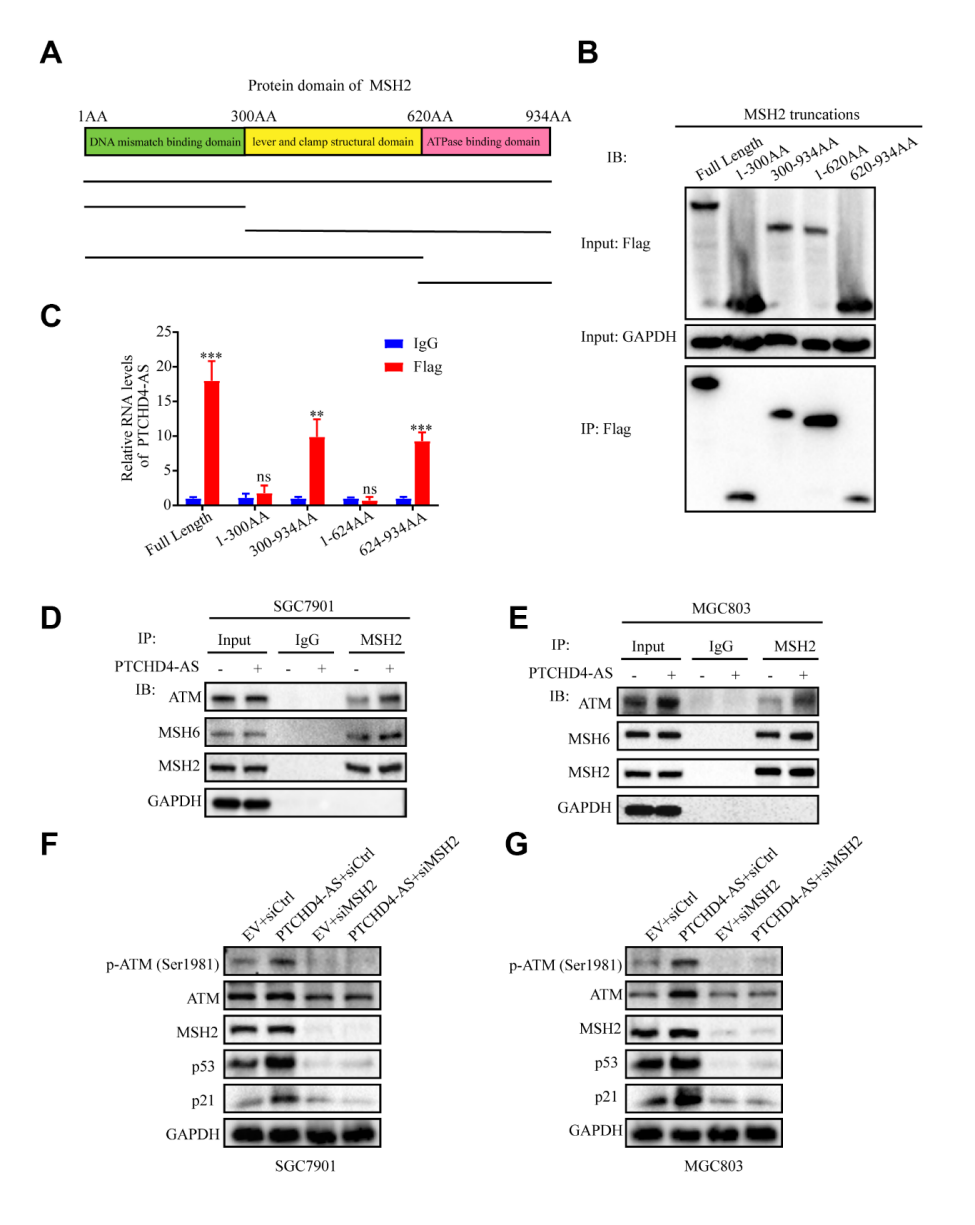
**Upregulation of PTCHD4-AS promoted MSH2-MSH6 dimerization and activated the ATM-p53-p21 pathway.** (**A**) Schematic diagram of truncated MSH2 used in pull-down assays. (**B**) After transfection of 3xFlag-MSH2 truncation in 293T cells for 48h, RIP was performed with anti-flag antibody. Western blot analyses of pull-down assays with truncated MSH2 fragments. (**C**) The relative expression level of PTCHD4-AS in the truncated MSH2 RIP was detected by RT-qPCR. IgG was used as a negative control. (**D**, **E**) Interaction analysis between MSH2, MSH6 and ATM in SGC7901 and MGC803 cells stably overexpressing EV or PTCHD4-AS. Co-IP experiments were performed with anti-MSH2 antibody, and Western blot was used to detect the expression of specific proteins in the precipitates. (**F**, **G**) Western blotting analysis of specific protein levels in SGC7901 and MGC803 cells stably overexpressing EV or PTCHD4-AS after transduction with siCtrl or siMSH2 for 48 h. Data are presented as mean ± SD, * *P* < 0.05, ** *P* < 0.01, *** *P* < 0.001.

Our findings demonstrated that the PTCHD4-AS molecule effectively suppressed the proliferation of GC cells through its interaction with the MSH2-MSH6 dimer. Nevertheless, the impact of PTCHD4-AS on the protein expression of MSH2 and MSH6 was not seen (Figure 4A), indicating that it might functionally regulate it. In light of the fact that the MSH2-MSH6 dimer is assembled through the ATPase domain of MSH2, we aimed to examine the potential interaction between PTCHD4-AS and this specific domain. The MSH2 protein encompasses three distinct domains in sequential order from its N-terminal to C-terminal regions. These domains include the N-terminal DNA mismatch repair binding domain (amino acids 1 to 300), the lever and clamp structural domain (amino acids 300 to 620), and the ATPase structural domain (amino acids 620 to 934) (54). We generated truncated MSH2 constructs according to these domains (Figure 5A) and performed flag-RIP assay using anti-flag antibody. We found that PTCHD4-AS bound to the ATPase domain of MSH2 (Figure 5B, 5C), indicating that it may facilitate the MSH2-MSH6 dimerization.

Previous studies have reported that the MSH2-MSH6 dimer could bind to and phosphorylate ATM, a key kinase in the DNA damage response pathway [38]. Moreover, activated ATM could phosphorylate p53 and activate its downstream target p21, finally leading to cell cycle arrest [39, 40]. Hence, we hypothesized that PTCHD4-AS might enhance the MSH2-MSH6 dimerization and promote its interaction with ATM, which in turn activates the ATM-p53-p21 pathway. To test this hypothesis, we performed a coIP assay using anti-MSH2 antibody in GC cells overexpressing PTCHD4-AS or empty vector. It was found that more MSH6 and ATM were co-precipitated with MSH2 in PTCHD4-AS overexpressing cells than in control cells (Figure 5D, 5E). Furthermore, it was found that PTCHD4-AS overexpression increased the expression of phosphorylated ATM, p53 and p21. These effects were reversed by MSH2 knockdown (Figure 5F, 5G). In addition, these results suggested that PTCHD4-AS possibly scaffold MSH2 and MSH6 and activated the ATM-p53-p21 pathway by enhancing the MSH2-MSH6 dimerization.

### PTCHD4-AS enhanced the sensitivity of GC cells to cisplatin via MSH2-MSH6 dimer

**Figure 6:**
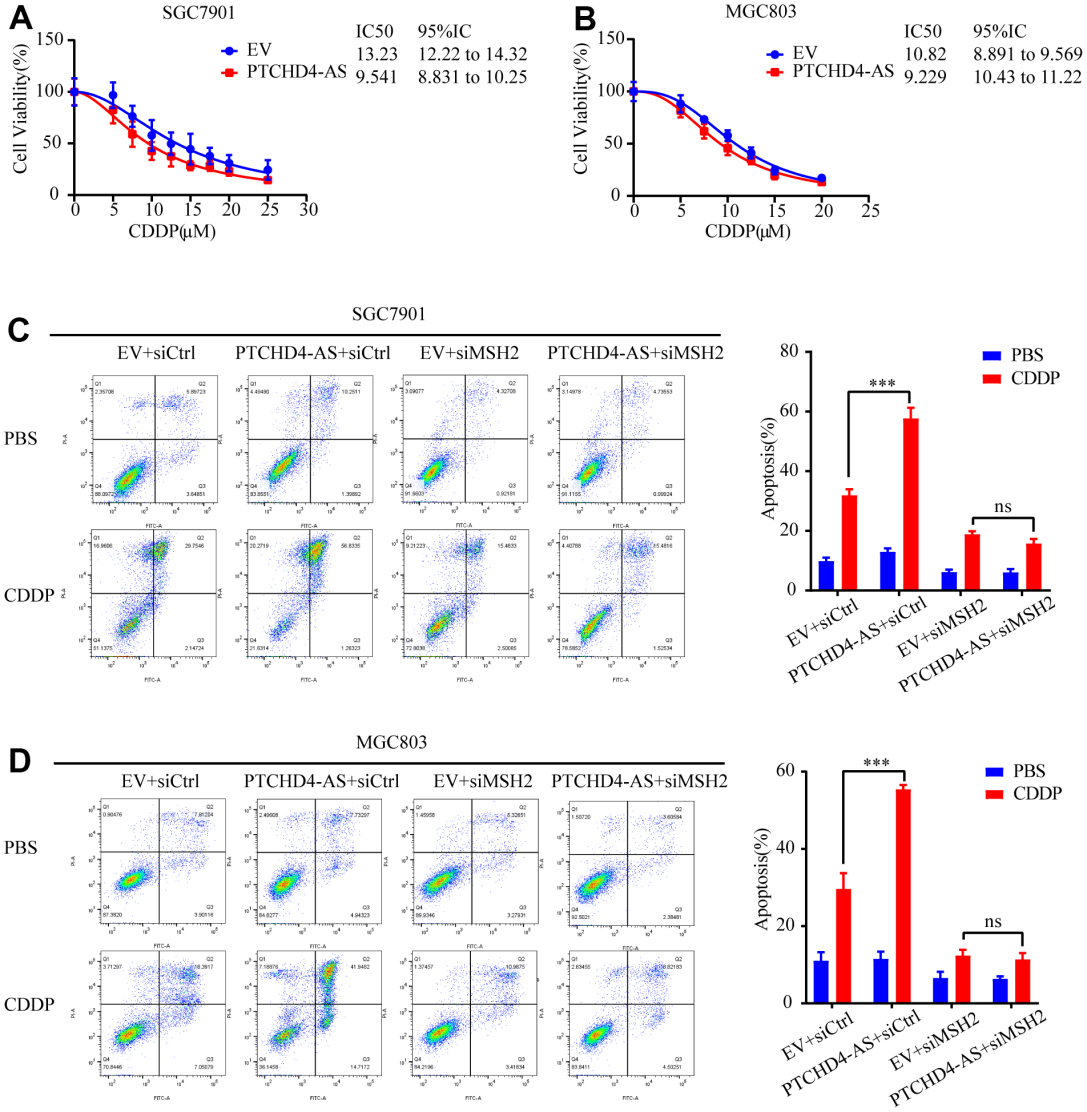
**PTCHD4-AS enhanced the sensitivity to CDDP by MSH2-MSH6 dimer in GC cells.** (**A**, **B**) Viability of SGC7901 and MGC803 cells stably overexpressing EV or PTCHD4-AS after treatment with the indicated concentrations of CDDP for 24 h. (**C**, **D**) Cell apoptosis was detected by FACS in SGC7901 and MGC803 cells stably overexpressing EV or PTCHD4-AS after transfection of siRNA for 48h, followed by treatment with CDDP or PBS for 24 h. Data are presented as mean ± SD, * *P* < 0.05, ** *P* < 0.01, *** *P* < 0.001.

Cisplatin (CDDP) is one of the most commonly used first-line chemotherapy drugs for GC [41]. However, its efficacy is often limited by drug resistance, which has been associated with impaired MMR function [42, 43]. Hence, we assessed the potential effect of PTCHD4-AS on the cellular response of GC cells towards cisplatin. It was observed that the overexpression of PTCHD4-AS resulted in decreased IC50 of CDDP in both SGC7901 and MGC803 cells (Figure 6A, 6B). Furthermore, we determined that the overexpression of PTCHD4-AS alone did not substantially induce apoptosis in GC cells. However, it did improve the apoptotic effect of CDDP. The observed effect was reversed after MSH2 knockdown (Figure 6C, 6D). These findings suggest that PTCHD4-AS enhances the sensitivity to CDDP via the MSH2-MSH6 dimer.

### PTCHD4-AS inhibited GC growth and increased cisplatin sensitivity *in vivo*

**Figure 7:**
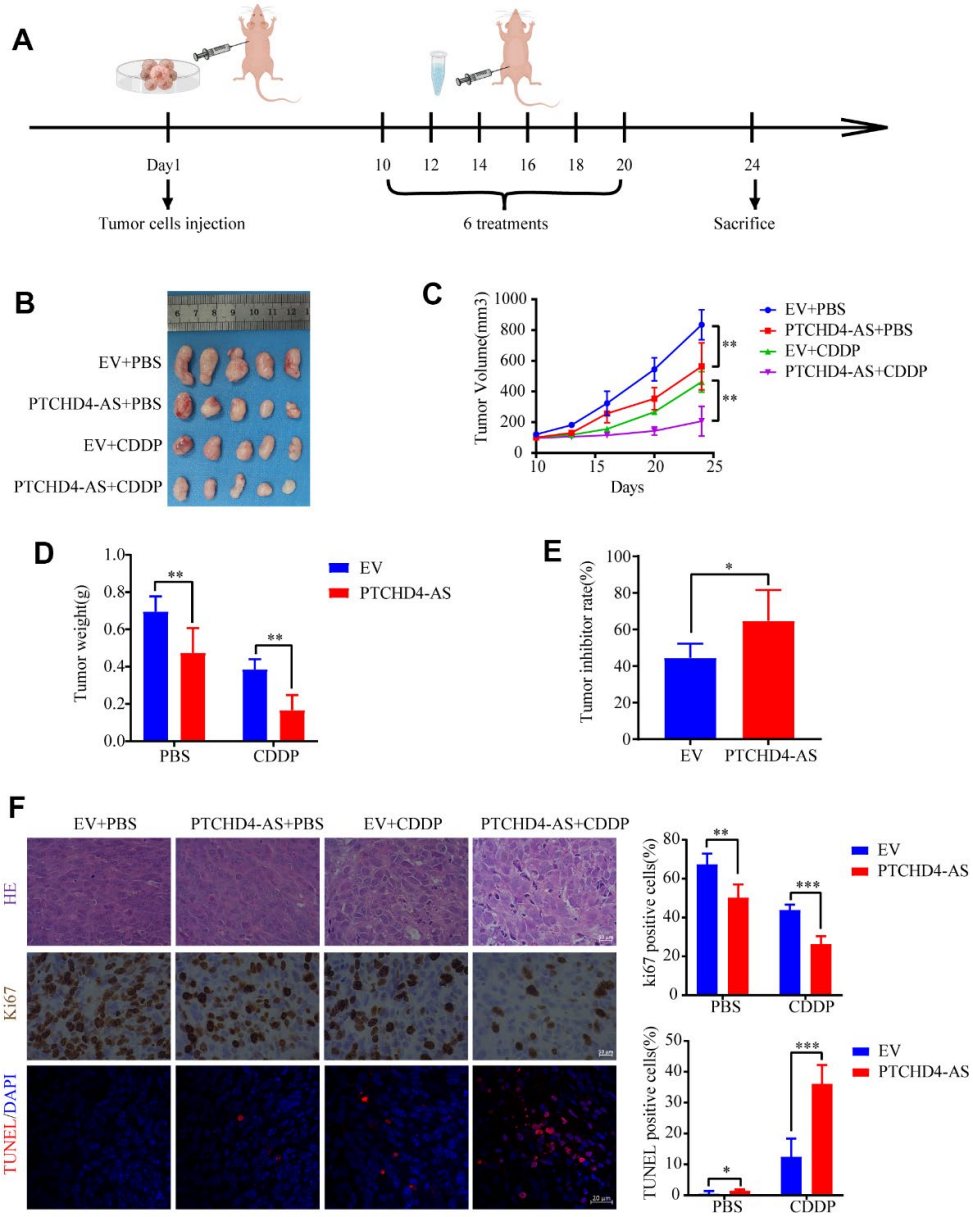
**PTCHD4-AS inhibited GC growth and increased cisplatin sensitivity *in vivo*.** (**A**) Schematic diagram of the xenograft tumor model of GC. (**B**) Images of xenograft tumors in different treatment groups at the end of the experiment. (**C**) Tumor growth curves of different treatment groups. (**D**) Tumor weights of different treatment groups at the end of the experiment. (**E**) Tumor inhibition rate of CDDP between EV and PTCHD4-AS groups. (**F**) Representative images and proportion of positive cells of Ki67 detected by IHC and apoptotic cells detected by TUNEL. Data are presented as mean ± SD, * *P* < 0.05, ** *P* < 0.01, *** *P* < 0.001.

In order to comprehensively determine the tumorigenic function of PTCHD4-AS and its impact on cisplatin therapy *in vivo*, we developed a xenograft model by introducing SGC7901 cells that were stably transfected with either PTCHD4-AS or an empty vector (Figure 7A). The tumor development was significantly suppressed in the overexpressed PTCHD4-AS group compared to the control group (Figure 7E). Immunohistochemical staining of tumor samples revealed that PTCHD4-AS overexpression inhibited Ki67 production, a standard assay for measuring cell proliferation. This effect was observed regardless of whether cisplatin therapy was administered or not. In contrast, the TUNEL assay demonstrated that the overexpression of PTCHD4-AS resulted in an elevated ratio of apoptotic cells within tumors that were subjected to cisplatin treatment (Figure 7F). The results suggested that PTCHD4-AS exerts inhibitory effects on the growth of and improves the sensitivity of GC cells to cisplatin treatment *in vivo*.

## DISCUSSION

The evolutionary conservation is frequently utilized as a means to evaluate the regulatory significance of recently discovered genes [44–46]. For example, Oskar et al. identified Pint, a widely expressed mouse lncRNA, as a direct p53 transcriptional target. Additionally, it was shown that the p53 protein controlled the human counterpart of PINT. Furthermore, the expression of PINT was found to be reduced in cases of colorectal cancer, whereas increased expression of PINT was observed to hinder the proliferation of tumor cells [47]. Subsequent investigations have provided additional evidence indicating that long non-coding RNA PINT has a regulatory influence on the proliferation of tumor cells and their sensitivity to radiochemotherapy across a range of malignancies [48], including gastric cancers [49], nasopharyngeal cancers [50], and skin cancers [51], and serves as a diagnostic/prognostic marker of cancer [52, 53].

In this study, we found a novel lncRNA PTCHD4-AS were conserved between humans and mice, as well as low expressed in GC tissue and cell lines generally. Notably, the transcription of lncRNA PTCHD4-AS was increased in response to DNA damage induction. Some DNA damage responsive lncRNAs, including lncRNA RoR [54], PANDA [55], and Meg3 [56] interact with different proteins involved in the cell cycle and apoptosis. We speculated that PTCHD4-AS might play a tumor suppressor role by participating in the DNA damage response. The study revealed that the overexpression of PTCHD4-AS led to the suppression of growth in GC cells by causing cell cycle arrest in the G2/M phase. Consistent with this, overexpression of PTCHD4-AS resulted in elevated levels of p53 and p21, which are the key proteins involved the cell cycle.

We further found that PTCHD4-AS binds with MSH2-MSH6 dimer which are essential proteins in DNA mismatch repair (MMR) [57]. It was found that cancer cells with MMR activation can lead to cell cycle arrest in the G2 phase, while MMR deficiency leads to reduced G2/M cell cycle arrest and reduced activation of the p53 pathway [32, 58]. To investigate whether MSH2-MSH6 dimer is responsible for the effect of PTCHD4-AS, we interfered with the expression of MSH2 and MSH6 by siRNA and found that it reversed the inhibition of cell growth and the arrest of cell cycle caused by upregulation of PTCHD4-AS. These data supported that PTCHD4-AS suppressed GC proliferation via MSH2-MSH6 dimer.

Then we investigated the effect of PTCHD4-AS on MSH2-MSH6 dimer and found that PTCHD4-AS did not directly affect the protein expression. Protein truncation experiments revealed that PTCHD4-AS binds to the ATPase structural domain of MSH2. Both MSH2 and MSH6 have a conserved ATPase structural domain, which also serves as a binding site for the dimer, and mutations in the binding site can inhibit MMR activation *in vivo* and *in vitro* [59, 60]. In other words, the ATP-binding domains are required to bind to each other to form an active dimer. In addition, MMR system acts as a molecular scaffold at DNA damage sites and promotes the activation of DNA damage pathway-associated kinases [32, 35], such as MSH2 can bind and activate ATM by phosphorylating it [38]. Therefore, we hypothesized that PTCHD4-AS regulates cell proliferation by affecting the activity of MSH2-MSH6 dimer. We confirmed by coIP assay that PTCHD4-AS promotes MSH2-MSH6 complex formation and binding to ATM. Furthermore, we found that overexpression of PTCHD4-AS increased the expression of pATM, p53 and p21, which was reversed by disruption of MSH2. Thus, PTCHD4-AS may promote the formation of MSH2-MSH6 dimer through “scaffolding”, which activates the ATM-p53-p21 pathway.

CDDP is a first-line chemotherapeutic regimen that induces DNA damage and activates cell cycle checkpoints primarily through forming platinum-DNA polymers, leading to apoptosis of cancer cells [49, 61, 62]. It has been known that the MSH2- MSH6 dimer recognizes cisplatin, and its functional defects can enhance resistance to cisplatin [63]. In bladder cancer, patients with low MSH2 protein levels have poor overall survival on CDDP-based therapy [64]. Whereas activation of MMR in ovarian and colon cancer cells increases the sensitivity to chemotherapeutic agents [65]. Since overexpression of PTCHD4-AS can activate MMR by promoting MSH2-MSH6 dimerization, it may enhance the cytotoxicity of cisplatin to GC cells, which was experimentally confirmed in this study. The cell cycle is a crucial factor affecting chemotherapy sensitivity [66, 67]. While PTCHD4-AS overexpression leads to cell cycle arrest, DNA damage caused by chemotherapy drugs can increase the transcription of PTCHD4-AS, further amplifying this effect, thereby increasing the therapeutic effect. The study also provides *in vivo* evidence of the anti-tumor effect of PTCHD4-AS using a mouse xenograft model.

In summary, we found a novel conserved lncRNA PTCHD4-AS and its overexpression inhibits GC cell proliferation and enhances the sensitivity to cisplatin. Mechanistically, PTCHD4-AS overexpression activates ATM-p53-p21 pathway by binding to MSH2-MSH6 dimer. Our study enriches the fact that conserved lncRNAs PTCHD4-AS plays a crucial role in GC development and may potentially be used as a chemotherapeutic target.

## MATERIALS AND METHODS

### Cell and tissue samples

This work obtained human normal gastric epithelial cell line (GES-1) and GC cell lines (SGC7901, MGC803, MKN45, AGS and NCI-N87) from the National Collection of Authenticated Cell Cultures (Shanghai, China. All cell cultures were maintained in RPMI-1640 medium (Gibco, USA), supplemented with 10% FBS (Biological Industries, USA) and 1% penicillin-streptomycin (Gibco, USA). HEK 293T was purchased from Procell company (Wuhan, China) and maintained in high-sugar DMEM (Gibco, USA), supplemented with 10% FBS and 1% penicillin-streptomycin. All cells were cultured in a constant-temperature cell culture incubator at 37° C with constant supply of 5% CO_2_.

Paired GC tissue samples and corresponding adjacent non-cancerous gastric tissue samples were obtained from the Fifth Affiliated Hospital of Zhengzhou University. All patients did not receive any treatment, including chemotherapy, radiotherapy, or any other medical intervention before surgery, and were diagnosed according to pathological evidence, and informed written consent was obtained for this study. Experimental procedures and sample collection were approved by the ethical committee of the Fifth Affiliated Hospital of Zhengzhou University (KY2022048). We strictly followed the guidelines of the Helsinki Declaration of 1964 and its latest amendments.

### RNA isolation and RT-qPCR

Total RNA from tissues/cells was extracted and purified using TRIzol (Invitrogen, USA). RNA was reverse transcribed to cDNA using ReverTra Ace qPCR RT Kit (Toyobo, Japan). RT-qPCR was performed using the Roche Light cycler480II system (Rochel, Switzerland) and ChamQ Universal SYBR RT-qPCR Master Mix (vazyme, China). The relative expression level of the target genes was calculated by the 2^−ΔΔCt^ method against GAPDH. Supplementary Table 1 displays the primer sequences.

### Rapid amplification of cDNA ends (RACE)

RACE was conducted with SMARTer RACE 5/3’ Kit (Clontech, USA) according to the manufacturer’s instructions. Briefly, the first strand (cDNA) was reverse transcribed from total RNA using modified oligonucleotides (supplied in the kit). 3'- or 5'-RACE fragments were amplified using the first strand as the template using a gene-specific primer (GSP) synthesized according to a known sequence. PCR products of the RACE assay were separated using 2% agarose gels.

### RNA FISH

Cy3-labeled PTCHD4-AS probes were synthesized by RiboBio company (China). Fluorescent *in Situ* Hybridization Kit (RiboBio, China) was applied to hybridize the probes to cells following the manufacturer’s instructions. Briefly, cells grown on 15 mm round coverslips were sequentially fixed with 4% paraformaldehyde for 10 min, permeabilized with 5% TritonX-100 for 5 min, blocked with pre-hybridization Solution for 30 min at 37° C and co-incubated with hybridization solution containing FISH probes at 37° C overnight. Cells were then washed sequentially with hybridization Wash I, II and III. The coverslips are directly covered with DAPI-containing anti-fluorescence quencher. Images were captured using a confocal laser scanning microscope (Carl Zeiss, Germany).

### Isolation of cytoplasmic and nuclear RNA

The cytoplasm and nucleus components were isolated using a Nuclear and Cytoplasmic Protein Extraction Kit (Beyotime, China) and RNase Inhibitor (Beyotime, China). Briefly, after cell collection, 200 μL of Cytoplasmic Protein Extraction Reagent A and 10 μL of RNase Inhibitor per 20 μL of cell sediment were added and ice bathed for 15 min. Then, we added 10 μL of Cytoplasmic Protein Extraction Reagent B and incubated on ice for 1 min. Then centrifuged at 12,000g for 5 mins at 4° C and supernatant was separated and precipitated completely, then added Trizol to extract cytoplasmic and nuclear RNA. The expression of PTCHD4-AS was determined by RT-qPCR. GAPDH and U6 were used as markers in the cytoplasm and nucleus, respectively.

### Plasmid construction and transfection

The entire length of PTCHD4-AS was cloned into pLenti6-puro vector. Lentiviral particles were prepared in HEK293T cells after transfecting with psPAX2, pMD2.G, and plasmids with a ratio of 4:3:1, and cell-free culture supernatants were used to infect gastric cancer cells. Cells with stable expression of PTCHD4-AS and negative control were selected with 4μg/ml of puromycin (Invitrogen).

Truncated segments of MSH2 were amplified with primers and subcloned into c-Flag pcDNA3 (Addgene plasmid#20011). MSH2 RNAi were purchased from Genepharma company (Shanghai, China). Both truncated MSH2 and siRNA were transfected using Lipo2000 (Invitrogen, USA) according to the manufacturer’s instructions. Protein and RNA were harvested after transfection (48 h).

### Cell proliferation assay

For CCK8 assays, ~2000 cells were seeded in a 96-well plate and incubated for indicated time, meanwhile 10 μL of CCK8 reagents was added. Cell viability was determined by Cell Counting Kit-8 (Meilunbio, China), and the absorbance at 450 nm was measured by (Bio-Rad, USA).

~500 cells were seeded in six-well plates for colony-formation assays and incubated for 10-14 days. The colonies were fixed with 4% paraformaldehyde (Biosharp, China), stained with 0.5% crystal violet (Beyotime, China), and photographed.

For the EdU assay, ~5x10^4^ cells were seeded in a six-well plate, incubated for 48 h, and then incubated with 10 μM EdU for 2 h. Cell viability was detected by EdU Cell Proliferation Kit with Alexa Fluor 488 (Meilunbio, China) according to the manufacturer’s instructions.

### Cell apoptosis assays

Cell apoptosis assays were carried out using the Annexin V-FITC/PI Apoptosis Detection Kit (KeyGEN, China). Cisplatin was dissolved in PBS. Cells were seeded into a six-well plate with/without cisplatin treatment. Apoptosis was measured and observed by flow cytometry using the BD FACS Aria™ III (BD Biosciences, USA). Data were analyzed by FlowJo software.

### Cell cycle assays

Cell cycle assays were conducted using the Cell Cycle Staining Kit (Multi Science, China). The cell cycle was measured and observed by flow cytometry using the BDAccuriC6 (BD Biosciences, USA). Data were analyzed by Modfit LT software.

### Western blotting

Whole cell lysates were made using RIPA buffer (Epizyme Biotech, China) containing protease inhibitor (Thermo Fisher, USA) and phosphatase inhibitor (Beyotime, China). The concentrations of protein were evaluated by BCA kit (Thermo Fisher, USA), mixed with SDS loading buffer, and boiled at 100° C for 10 min. The protein samples were subjected to SDS PAGE and transferred to nitrocellulose membranes. The membranes were blocked using a 5% solution of non-fat milk in TBST for 1 hour at room temperature. Subsequently, the membranes were incubated overnight at 4° C with primary antibodies. The primary antibodies used were as follows: p53 anti-mouse (ab137797, Abcam, UK), p21(10355-1-AP, Proteintech, USA), MSH2 Polyclonal antibody (15520-1-AP, Proteintech, USA), MSH6 (18120-1-AP, Proteintech, USA), ATM (27156-1-AP, Proteintech, USA), Phospho-ATM (Ser1981) (5883, CST, USA), Flag (66008-4-Ig, Proteintech, USA), β-tubulin (10094-1-AP, Proteintech, USA) and GAPDH (60004-1-Ig, Proteintech, USA). After washing with TBST, the membranes were incubated with HRP-conjugated secondary goat anti-mouse (ZB2305) or goat anti-rabbit (ZB-2301) antibodies (ZSGB-BIO, China) or HRP-goat anti-mouse IgG LCS (AMJ-AB2016, Abbkin, China) for 1 h at room temperature. Protein bands were visualized using enhanced chemiluminescence (Epizyme Biotech, China).

### RNA pull-down assay and RNA binding protein immunoprecipitation assay (RIP)

For RNA pull-down, biotin-labeled lncRNA probes and control probes against PTCHD4-AS were synthesized by General Biotechnology (Anhui, China). All solutions were prepared in DEPC water. In short, the probes were incubated with the streptavidin Dynabeads (MCE, USA) at 4° C overnight. The next day, the fresh cells were collected and lysed with IP buffer (25 mM Tris-HCl 150 mM NaCl 1 mM EDTA 1% NP-40) supplemented with RNase Inhibitor (MCE, USA) and protease inhibitor cocktail (PIC) on ice for 30 min, then centrifuged at 12,000 rpm for 20 min and the supernatants were collected. The supernatants were incubated with the probe-beads complex at 4° C for 3 h. The beads were resuspended in SDS loading buffer (Epizyme Biotech, China) after being rinsed with IP solution 5 times. Boiling at 100° C for 10 min, eluted the proteins bound to the probes, which SDS-PAGE then separated. Nevertheless, the examination of the gel bands was conducted utilizing mass spectrometry. The sequences of the probes are available in Supplementary Table 1.

For RIP, anti-MSH2, anti-MSH6 antibody, and IgG were incubated with protein A/G magnetic beads overnight at 4° C. Cells were lysed, centrifuged, incubated with magnetic beads, and eluted as described above. Finally, RNA was isolated and purified using phenol-chloroform extraction, and the relative enrichment of PTCHD4-AS was analyzed by RT-qPCR.

### Co-immunoprecipitation (coIP)

The specified antibodies or IgG control were incubated overnight at 4° C with protein A/G beads (MCE, USA). Fresh cells were lysed in IP buffer supplemented with protease inhibitor cocktail (PIC) for 20 min on ice. Cell lysates were incubated with different antibody-beads complexes for 6 h at 4° C before washing 5 times in IP buffer. The proteins attached to the antibodies were extracted by boiling at a temperature of 100° C for 10 min. Subsequently, these proteins were separated and visualized using SDS-PAGE. The protein levels were evaluated using the western blotting technique.

### Animal experiment

Female BALB/c nude mice aged 3-4 weeks were purchased from Huafukang Biotechnology (Beijing, China) and acclimatized in the SPF-grade standard animal house of the Fifth Affiliated Hospital of Zhengzhou University for one week. About 5×10^6^ SGC7901 cells stably expressing PTCHD4-AS and empty vector were injected subcutaneously into the back of the mice. Upon attaining a tumor volume of around 100 mm^3^, the mice were randomly allocated EV+PBS group, PTCHD4-AS+PBS group, EV+CDDP group, and PTCHD4-AS+CDDP group (n=5). CDDP (4 mg/kg) or an equivalent volume of PBS was injected intraperitoneally every two days six times. The tumor length (a) and width (b) were measured twice a week using a caliper, and the tumor volume (V) was calculated as V(mm3) =1/2ab^2^. On day 24, mice were euthanized using anesthesia, and tumors were excised, weighed, and fixed in 4% paraformaldehyde (Biosharp, China).

### HE, IHC and TUNEL

Hematoxylin and eosin (HE) staining, Ki67 immunohistochemistry (IHC), and terminal deoxynucleotidyl transferase-mediated dUTP nick end labeling (TUNEL) test were performed on paraffin-embedded tumor sections. Ki67 was evaluated by IHC using the SPlink Detection Kit (ZSGB-BIO, China) after sections were stained with HE-staining kit (Beyotime, China). To do this, we used Ki67 (ab15580; Abcam, UK) as our main antibody. The TUNEL test was performed using the *In Situ* Cell Death Detection Kit (Beyotime, China), while ImageJ was used to quantify how many cells stained positive for Ki67 or TUNEL.

### Statistical analysis

All of the statistical analyses were performed using Prism 8 (GraphPad, USA). The non-parametric the Wilcoxon test was employed to compare the samples of human subjects to accommodate non-normally distributed data. Student’s t-test (unpaired, two-tailed) was used to assess differences between two groups. One-way analysis of variance (ANOVA) was used to assess differences between three or more groups. *P* < 0.05 was chosen as the level of statistical significance. Asterisks denote significant differences (*P < 0.05, **P < 0.01, ***P < 0.001). All tests were performed in triplicate, and the results are shown as the mean, standard deviation to show both the average and the range of results.

### Data availability statement

The data from the study are included in the supplementary material, and the authors should be contacted for further consultation.

### Consent statement

Informed consent was obtained from all subjects involved in the study.

## Supplementary Material

Supplementary Figures

Supplementary Tables
