# Supplementary material for: Lnc-PTCHD4-AS inhibits gastric cancer through MSH2-MSH6 dimerization and ATM-p53-p21 activation
Source: Aging (Albany NY). 2023 Nov 27;15(22):13558–78. doi: 10.18632/aging.205329 (PMC10713396; doi:10.18632/aging.205329)
Supplement: Supplementary Figures [file aging-15-205329-s001.pdf]

SUPPLEMENTARY FIGURES

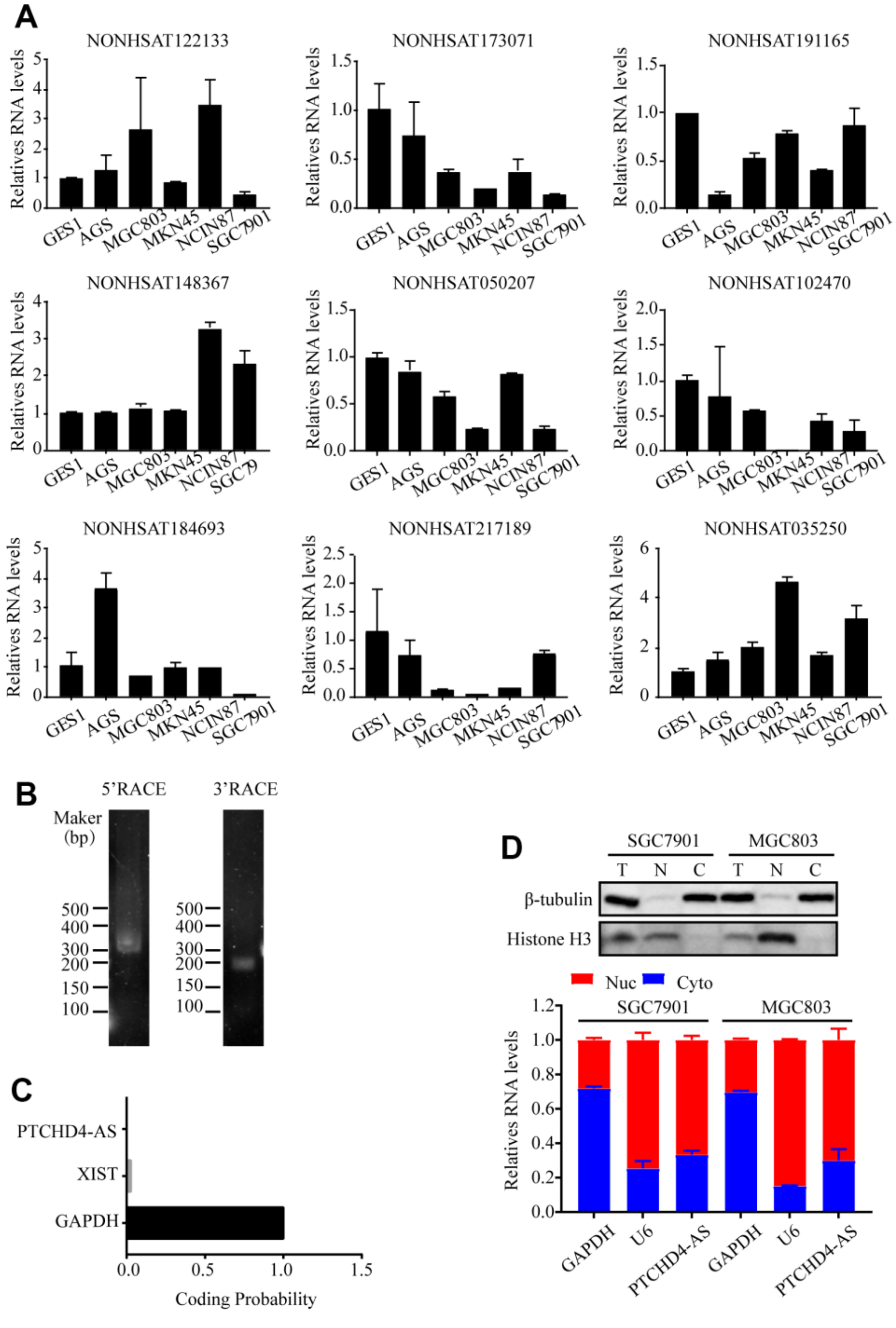

Supplementary Figure 1. Identification and characterization of PTCHD4-AS. (**A**) Relative expression levels of screened conserved lncRNAs (top9) in normal gastric epithelial cell lines and GC cell lines. (**B**) Agarose gel electrophoresis showed that the PCR products of 5' and 3' RACE of PTCHD4-AS. (**C**) The protein coding ability of PTCHD4-AS was predicted by CPAT; the protein coding gene GAPDH and the non-coding gene Xist were used as positive control and negative control, respectively. (**D**) Western blots of total cell lysates (T), nucleus fraction (N) and cytoplasm fraction (**C**), β-tubulin and histone H3 as the marker of the nucleus and cytoplasm, respectively (upper). Relative expression of PTCHD4-AS in the nucleus and cytoplasm fractions by RT-qPCR. GAPDH and U6 were used as positive controls for cytoplasm and nucleus, respectively. Data are presented as mean ± SD, * $P < 0.05$, ** $P < 0.01$, *** $P < 0.001$.

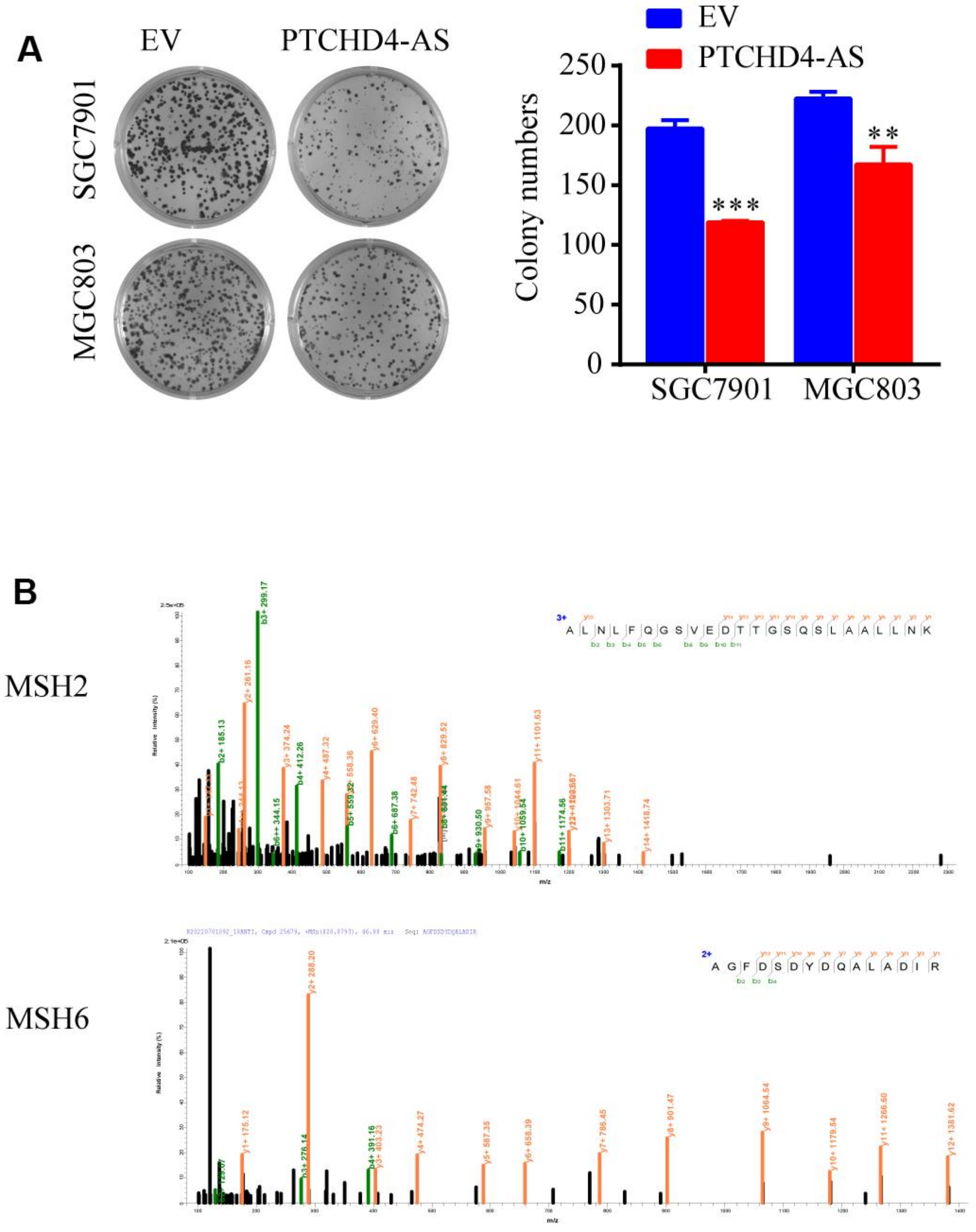

Supplementary Figure 2. PTCHD4-AS suppress GC cell growth *in vitro.* (**A**) Representative images and statistical analysis of colony formation in SGC7901 and MGC803 cells stably expressing EV or PTCHD4-AS. (**B**) The secondary mass spectra of MSH2 and MSH6.
