# Supplementary material for: Lnc-PTCHD4-AS inhibits gastric cancer through MSH2-MSH6 dimerization and ATM-p53-p21 activation
Source: Aging (Albany NY). 2023 Nov 27;15(22):13558–78. doi: 10.18632/aging.205329 (PMC10713396; doi:10.18632/aging.205329)
Supplement: Supplementary Tables [file aging-15-205329-s002.pdf]

SUPPLEMENTARY TABLES

Supplementary Table 1. Sequence of primers and probes.

Gene	Primer	Sequence (5′-3′)
		**Primers for qPCR**
NONHSAT122133	Forward	TGCTGAAACTTGATAGAAATG
	Reverse	TCCTCAATACTTTGAGTTGT
NONHSAT173071	Forward	CAGATGTTGGCTTTGTAATT
	Reverse	TTGTCTACGCTTCACAGAG
NONHSAT191165	Forward	AATCTGTCAATCCTGTCCGT
	Reverse	AAACTTAAAGGAATTGACGGA
NONHSAT148367	Forward	GAATTCTTCACCCAAAACTC
	Reverse	TCATTTATGCCTCTGCTATT
NONHSAT050207	Forward	AACATGACATCGGGAAGTCC
	Reverse	AGTGGGGTTATTCATTAGAG
NONHSAT102470	Forward	ACTGCTCGGGCTGAATAGCA
	Reverse	CACAACTTCCGGGATAATGAGA
NONHSAT184693	Forward	CAGAACCACACACTATGTAA
	Reverse	ATCACAGCCCATATTGAGAG
NONHSAT217189	Forward	ACTCTCTTAAGGTAGCCAAA
	Reverse	TTTCGCTGGATAGTAGGT
NONHSAT035250	Forward	ATGCTGGCTCCCATTCAGC
	Reverse	TGCGTCGCTTTTGGCTCC
PTCHD4-AS	Forward	ACGTAAAGAGAGTATGGAGCTCGT
	Reverse	TGTTCCAGAGGGTGCTTTCCA
GAPDH	Forward	GTCTCCTCTGACTTCAACAGCG
	Reverse	ACCACCCTGTTGCTGTAGCCAA
U6	Forward	GCTTGCTTCAGCAGCACATA
	Reverse	AAAAACATGGAACTCTTCACG
MSH2	Forward	GTCACAGCACTCACCACTGAAG
	Reverse	AAGCTCTGCAACATGAATCCCA
MSH6	Forward	TCCACCAGATACTCCTCAACAACT
	Reverse	GCACTTCCAACGTACTCCTTGC
		**Primers for RACE**
3'RACE		CACCTTGGGCAAGTTTGCTCTCTG
5'RACE		CCTTGGGTCATCTCATTTGATTCCC
		**Primers for plasmid construction**
PTCHD4-AS	Forward	AGAAGACACCGACTCTAGAGGATCCCTCAGCCTGGAGCAAAATAT
	Reverse	TCCAGAGGTTGATTATCGATCTCGAGAAGGTGTTGCGAGGAAATC
MSH2 FL	Forward	CTTAAGCTTGGTACCGGATCCATGGCGGTGCAGCCGAAG
	Reverse	ATCATCCTTGTAATCTCTAGACGTAGTAACTTTTATTCGTG
MSH2 1-300aa	Forward	CTTAAGCTTGGTACCGGATCCATGGCGGTGCAGCCGAAG
	Reverse	ATCATCCTTGTAATCTCTAGAATACTGGCTGAAGTCAAAAG
MSH2 300-934aa	Forward	CTTAAGCTTGGTACCGGATCCATGAAATTGGATATTGCAGCA
	Reverse	ATCATCCTTGTAATCTCTAGACGTAGTAACTTTTATTCGTG
MSH2 1-620aa	Forward	CTTAAGCTTGGTACCGGATCCATGGCGGTGCAGCCGAAG
	Reverse	ATCATCCTTGTAATCTCTAGAATATGGAACAGGTGCTCCAT
MSH2 620-934aa	Forward	CTTAAGCTTGGTACCGGATCCATGGTACGACCAGCCATTTTGG
	Reverse	ATCATCCTTGTAATCTCTAGACGTAGTAACTTTTATTCGTG

		Probes for RNA pull down
Control	probe 1	BIOTIN-CGCTGAGGCCGAAGGTGATTGTCAGG
	probe 2	BIOTIN-GACATGTGTAGGATGATGCTGCA
PTCHD4-AS	probe 1	BIOTIN-CCTGACAATCACCTTCGGCCTCAGCG
	probe 2	BIOTIN-TGCAGCATCATCCTACACATGTC

Supplementary Table 2. Conservative top 10 lncRNAs.

NONCODE TRANSCRIPT ID	Identity (%)	Alignment length(nt)	Gap openings	Start	End	e-value	Bit score
NONHSAT122133	99.583	480	0	48767	49246	0	876
NONHSAT173071	99.475	381	0	4422	4802	0	693
NONHSAT191165	99.35	923	0	4117	5039	0	1672
NONHSAT148367	98.71	310	0	19097	19406	2.47E-153	551
NONHSAT050207	98.621	290	0	7072	7361	2.72E-143	514
NONHSAT102470	98.502	601	0	1840	2440	0	1061
NONHSAT184693	98.305	354	0	58087	58440	1.24E-174	621
NONHSAT217189	97.659	299	0	46526	46824	1.93E-142	514
NONHSAT035250	97.583	331	0	44390	44720	1.43E-158	568
NONHSAT207347	97.494	399	0	243923	244321	0	682

Notes:

1. identity (%): The percentage of agreement for sequence alignment.
2. alignment length: The length of the alignment area that conforms to the alignment.
3. Gap openings: The number of gaps in the comparison area.
4. start: the starting site of the alignment region on the human genome.
5. end: the end site of the alignment region on the human genome.
6. e-value: The smaller the value, the higher the confidence. 7. bit score: The higher the value, the higher the credibility

The code is as follows:

```
grep “>” file.fa | wc -l
makeblastdb -in outLncRNA.fa -dbtype nucl
blastn -query mouse_lncrna.fasta -db outLncRNA.fa -outfmt 6 -out blastn2.out
```
